# *Gallid herpesvirus* 3 SB-1 strain as a recombinant viral vector for poultry vaccination

**DOI:** 10.1038/s41541-018-0056-6

**Published:** 2018-05-28

**Authors:** Yashar Sadigh, Claire Powers, Simon Spiro, Miriam Pedrera, Andrew Broadbent, Venugopal Nair

**Affiliations:** 1 The Pirbright Institute, Ash Road, Woking, GU24 0NF United Kingdom; 2 Kennedy Institute of Rheumatology, University of Oxford, Roosevelt Drive, Headington, Oxford, OX3 7FY United Kingdom; 3 Department of Pathobiology and Population Sciences, The Royal Veterinary College, Hawkshead Lane, Hatfield, AL9 7TA United Kingdom

## Abstract

Live herpesvirus-vectored vaccines are widely used in veterinary medicine to protect against many infectious diseases. In poultry, three strains of herpesvirus vaccines are used against Marek’s disease (MD). However, of these, only the herpesvirus of turkeys (HVT) has been successfully developed and used as a recombinant vaccine vector to induce protection against other avian viral diseases such as infectious bursal disease (IBD), Newcastle disease (ND) or avian influenza (AI). Although effective when administered individually, recombinant HVT vectors have limitations when combined in multivalent vaccines. Thus there is a need for developing additional viral vectors that could be combined with HVT in inducing protection against multiple avian diseases in multivalent vaccines. *Gallid herpesvirus* 3 (GaHV3) strain SB-1 is widely used by the poultry industry as bivalent vaccine in combination with HVT to exploit synergistic effects against MD. Here, we report the development and application of SB-1 as a vaccine vector to express the VP2 capsid antigen of IBD virus. A VP2 expression cassette was introduced into the SB-1 genome at three intergenic locations (UL3/UL4, UL10/UL11 and UL21/UL22) using recombineering methods on the full-length pSB-1 infectious clone of the virus. We show that the recombinant SB-1 vectors expressing VP2 induced neutralising antibody responses at levels comparable to that of commercial HVT-based VAXXITEK_HVT+IBD_ vaccine. Birds vaccinated with the experimental recombinant SB-1 vaccine were protected against clinical disease after challenge with the very virulent UK661 IBDV isolate, demonstrating its value as an efficient viral vector for developing multivalent vaccines against avian diseases.

## Veterinary medicine: an effective vaccine platform to overcome multiviral interference

Gallid herpesvirus 3 (GaHV3) strain SB-1 is effective in delivering vaccine material to cells, overcoming a major roadblock in poultry vaccination. UK researchers, led by the Prof. Venugopal Nair OBE at the Pirbright Institute, have constructed a vaccine against Marek's disease virus (MDV) and infectious bursal disease viruses (IBDV). Both MDV and IBDV are deadly viruses of poultry industry. For this purpose, GaHV3 strain SB-1 (a vaccine strain of virus against MDV) was used and genetic materials for VP2 (one of the surface glycoproteins of IBDV) was incorporated into the genome of SB-1. Cells inoculated with the viral vaccine produce the VP2 proteins of IBDV which then primes a host’s immune system response against infection. Birds immunized with the team’s vaccine remained free of clinical symptoms after IBDV infection, highlighting the potential of SB-1 to be used as a vector against other avian diseases. The use of SB-1 also sidesteps a constraint of previous vaccines, based on herpesvirus of turkeys (HVT), which is recommended not to be combined with other HVT based vaccines. SB-1 vaccines can be administered alongside HVT vaccines and they can provide a better immunity against MDV, highlighting the potential of SB-1 to be used as a vector against other avian vidal diseases.

## Introduction

Marek’s disease virus (MDV-1) or *Gallid herpesvirus* 2, belonging to the *Mardivirus* genus in the family *Herpesviridae*, is the causative agent of Marek’s disease (MD) characterised by rapid-onset lymphomatous tumours and paralytic symptoms due to neuronal infiltration of lymphocytes. In infected birds, MDV replicates in the feather follicle epithelial cells, from where it is transmitted to other birds by inhalation of infected dust and dander. The mortality rate from MD usually varies between 10–30%, but can be up to 100% dependent on infecting strain, host susceptibility and/or vaccination status. MD has been controlled for more than 4 decades by the widespread use of live attenuated vaccines.^1,2^ Vaccine strains include the naturally attenuated MDV-1 strain Rispens (CVI988), MDV-2 (GaHV3) strain SB-1 and herpesvirus of turkeys (HVT) strain FC126.

In addition to their use as successful vaccines inducing long-term protection against MD, avian herpesvirus vaccine strains also have the potential to be used as recombinant vaccine viral vectors for inducing protection against other major avian diseases. The most successful and widely-used recombinant herpesvirus vaccine vector is the HVT strain which has been shown to be highly effective in protecting against a number of avian viral pathogens,^3–6^ even in the presence of maternal antibodies.^7^ Although individual recombinant HVT vaccines have proven to be extremely effective, combined use of more than one recombinant HVT vaccine has been shown to be less so. This is thought to be due to the interference between different HVT vaccines, because of which manufacturers do not recommend the use of combinations of HVT-based vaccines.^8^ Thus the combined use of HVT to protect against MD and another recombinant HVT vectored vaccine to induce protection against a second disease, may lead to a failure. Generation of multivalent HVT vectors expressing multiple foreign genes have also been technically challenging. With these constraints on the use of HVT vectored vaccines, there is a need for developing other vector platforms that will not interfere, but rather complement the induction of protective responses against multiple antigens in the vaccine.

The first GaHV3 strain that was licensed for use as a vaccine against MD was the SB-1 strain.^9^ It was originally introduced in the mid-1980s and is still used very successfully in combination with the HVT vaccine, inducing protection against very virulent MDV pathotypes.^2^ Commercial bivalent vaccines containing SB-1 and HVT FC126 strains are widely used in many countries including USA, South America and Asia.^1,10^ SB-1/HVT bivalent vaccines are thought to provide superior protection against MD through a complementing effect^2,11^ even in maternal antibody-positive chicks.^11^ Because of the complementing effects of the HVT and SB-1 strains in inducing protection against MD, we reasoned that the SB-1 recombinant viral vector could be used as a bivalent vaccine in combination with recombinant HVT with complementary rather than inhibitory effects. We have previously shown that recombinant HVT expressing HA of AI virus was capable of producing immunity against MDV and AI lethal challenge.^4^

The 166-Kb genome of the SB-1 strain of GaHV3 has a similar genome organisation as MDV-1, sharing a number of homologous genes^12^ as well as unique set of genes and microRNAs.^13^ We have previously reported the construction of the full-length infectious genome of the SB-1 strain in a bacterial artificial chromosome (BAC) that could be used as a reverse genetics tool for manipulation of the viral genome.^14,15^ We have also shown that the virus reconstituted from the recombinant SB-1 BAC clone induced strong protection against virulent MDV challenge.^15^ This has allowed us to develop SB-1 as a recombinant vector for expressing protective antigens from other avian pathogens using the well-established recombineering techniques.^16^ Here we report the construction of a recombinant SB-1 viral vector engineered to express the VP2 capsid protein^17^ of infectious bursal disease virus (IBDV) to induce immune responses and protection against the very virulent UK661 IBDV isolate.

## Results

### Generation of SB-1-UL3/4VP2, SB-1-UL10/11VP2 and SB-1-UL21/22VP2 viruses

**Fig. 1:**
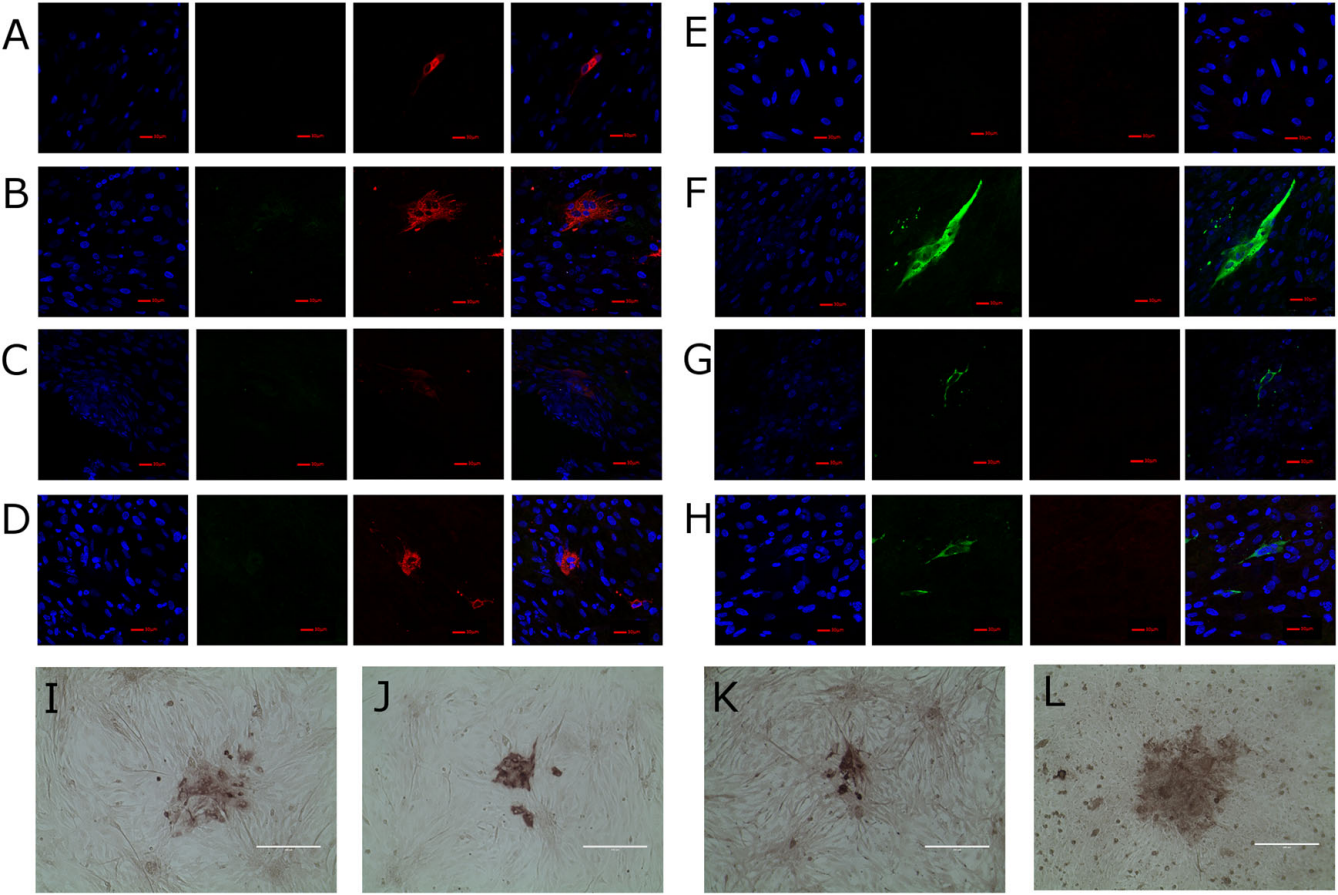
**a**–**d** Infected cells stained against SB-1 with anti gB Y5.9 antibody (IgG1) followed by Alexa Fluor 568 staining. **e**–**h** infected cells stained separately against IBDV VP2 with anti VP2 antibody HH7 (IgG1) followed by Alexa Flour 488 staining. The nuclei of the cells are stained with DAPI and shown in blue (scale bar = 30 µm). **a** and **e** show CEF cells infected with parental pSB-1 virus, **b** and **f** show CEF cells infected with pSB-1-UL3/4VP2 recombinant virus, **c** and **g** show CEF cells infected with pSB-1-UL10/11VP2 recombinant virus and **d** and **h** show CEF cells infected with pSB-1-UL21/22VP2 recombinant virus. **i**–**l** show plaque formation in CEF cells infected with pSB-1 (**i**), CEF cells infected with pSB-1-UL3/4VP2 (**j**), pSB-1-UL21/22VP2 (**l**) and pSB-1-UL10/11VP2 (**k**) recombinant viruses (scale bar = 200 µm)

**Fig. 2:**
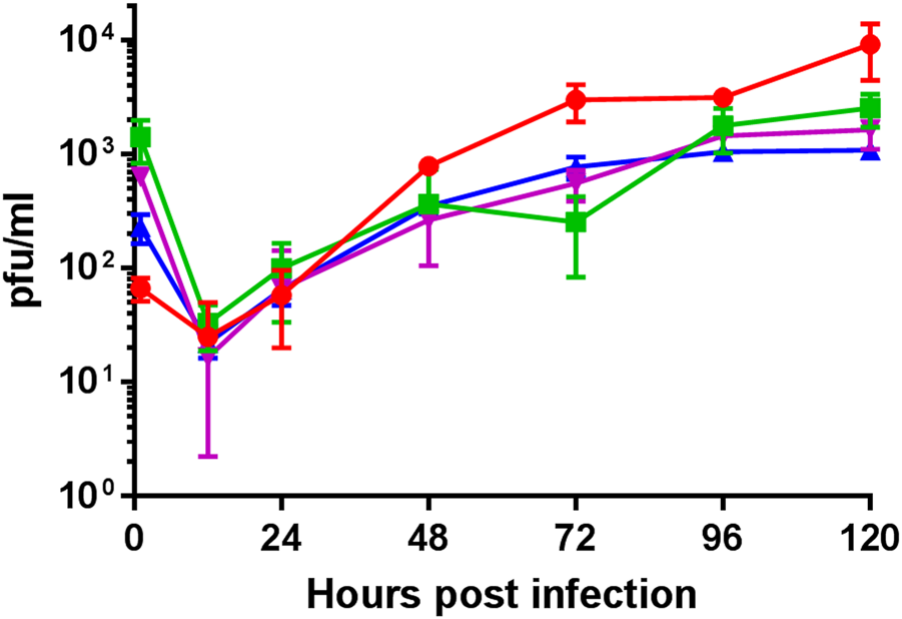
Growth curve for SB-1 recombinant viruses in vitro. Time course experiment was performed in triplicate with each time point was infected independently. Each replicate was titrated in duplicate. PFU in 1 ml of infected cell suspension was calculated for each time point; SB-1(), SB-1-UL3/4VP2 (), SB-1-UL10/11VP2 () and SB-1-UL21/22VP2 (). A significant difference at time points 72 and 120 h (*p* = 0.0031 and *p* = 0.0001, respectively) was observed for SB-1-UL3/4VP2 when compared to its parental virus. Similarly, a significant difference was observed between the titre of SB-1 and SB-1-UL21/22VP2 time points 72 and 120 h (*p* = 0.0094 and *p* = 0.0001, respectively). In comparison with the other two recombinant viruses, SB-1-UL10/11VP2 showed a significant difference at time points 72, 96 and 120 h post infection (*p* = 0.0194, *p* = 0.0284 and *p* = 0.0001, respectively)

The expression for IBDV VP2 was derived from a murine cytomegalovirus immediate early protein 2 (mCMV IE2) promoter and the VP2 gene was derived from IBDV strain Faragher 52/70 with a SV40 early polyadenylation signal (Venugopal Nair, unpublished data). Expression of VP2 in cells infected with recombinant SB-1 viruses was assessed by staining with the anti-IBDV VP2 mouse monoclonal antibodies clone HH7 (IgG1) (Fig. 1 Panels A to D). SB-1 infection was confirmed by staining with anti-gB antibody Y5.9 (IgG1)^18^ (Fig. 1, Panels E to H). Virus plaques were visualised by antibody staining followed by colour development using peroxidase (HRP) substrate (Fig. 1i for pSB-1, Fig. 1j for pSB-1-UL3/4VP2, Fig. 1k for pSB-1-UL21/22VP2 and Fig. 1l for pSB-1-UL10/11VP2). Recombinant SB-1 virus isolated from cells that had been transfected with the BAC DNA was passaged three times in primary chicken embryonic fibroblast (CEF) cells to produce virus stocks. Studies comparing the in vitro growth showed no significant differences between the parental pSB-1 virus and the recombinant SB-1-UL3/4VP2 viruses between 12 and 48 h post-infection (Fig. 2). All of the recombinant SB1 viruses showed reduced levels of replication at time points 72, 96 and 120 h post infection. Based on a two way ANOVA with multiple comparisons test, there is a significant difference between SB1 and SB-1-UL3/4VP2 growth rate at time points 72 and 120 h (*p* = 0.0031 and *p* = 0.0001, respectively). Likewise, at time points 72 and 120 h post infection, a significant difference was observed between pSB-1 and SB-1-UL21/22VP2 (*p* = 0.0094 and *p* = 0.0001, respectively). In comparison with the other two recombinant viruses, SB-1-UL3/4VP2 showed a significant difference at time points 72, 96 and 120 h post infection (*p* = 0.0194, *p* = 0.0284 and *p* = 0.0001, respectively). Overall, the replication rate for all three viruses were slower compared to the parental virus.

### Immune response to vaccination and protection against virulent infection

Considering that IBDV strain UK661 does not grow efficiently in DF-1 cells, we used the IBDV vaccine strain D78 to perform neutralisation assays. Usage of IBDV strain D78 gave us the opportunity to detect viral cytopathic effect on the adherent DF-1 cell line. The amino acid sequence of the VP2 gene from strain UK661 has 99% identity with strain F52/70 and 98% identity with strain D78. (GenBank accession numbers EU162087.1, NC_004178.1 and D00869.2).^19–21^

**Fig. 3:**
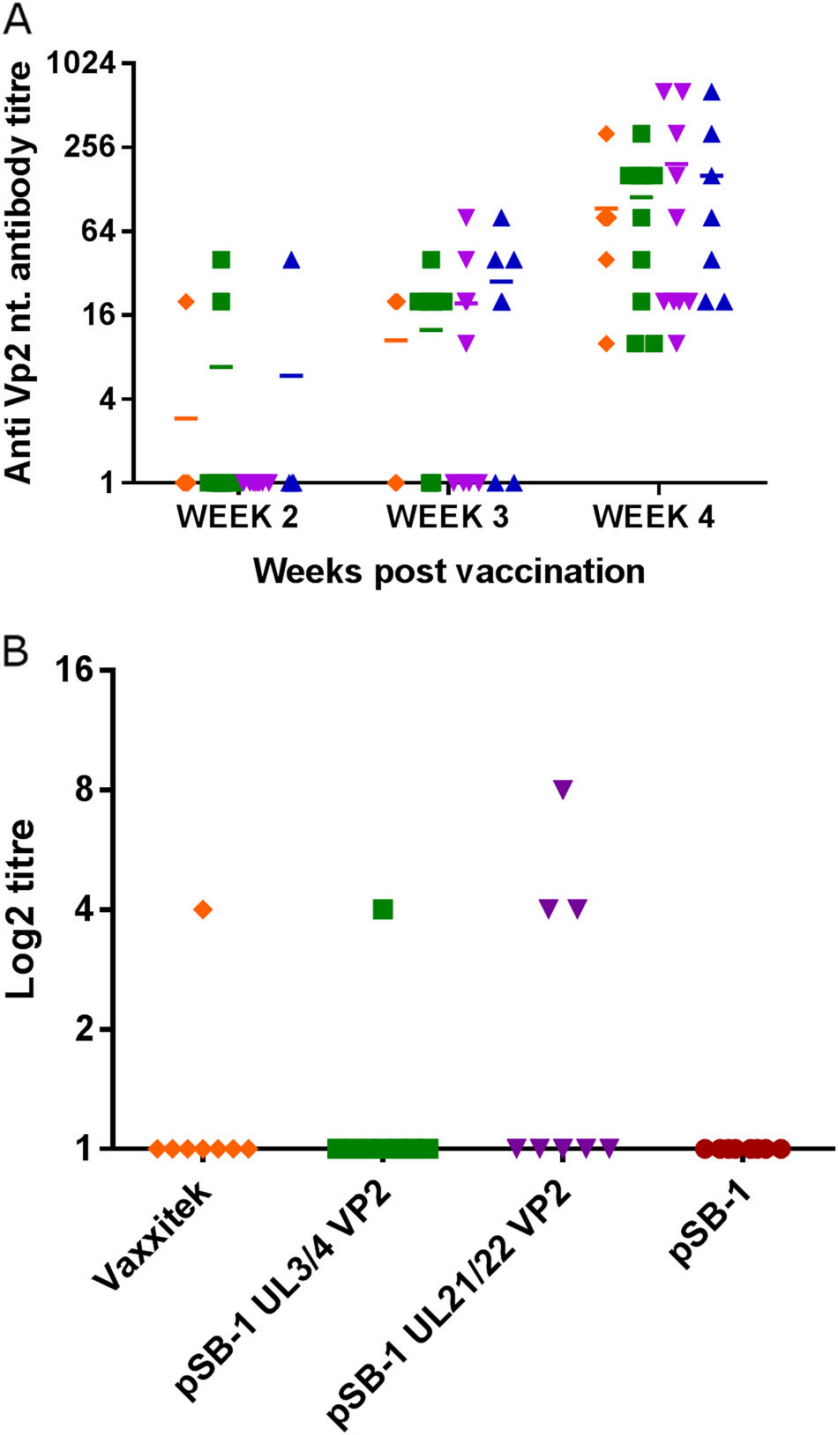
**a** Experiment 1—Titre of neutralising antibodies in the sera of chickens vaccinated with VAXXITEK_HVT+IBD_ (), SB-1-UL3/4VP2 (), SB-1-UL21/22VP2 () or SB-1-UL10/11VP2 () vaccine viruses. Serum samples were collected at weeks 2, 3 and 4 post-vaccination. The mean titre in each group is shown as a horizontal bar. **b** Experiment 2—Titre of neutralising antibodies in week 3 serum of chickens vaccinated with VAXXITEKHVT + IBD (), SB-1-UL3/4VP2 (), SB-1-UL21/22VP2 () or SB-1-UL10/11VP2 (). Individual values are shown. Group names are given on the *X*-axis

As it is shown in Fig. 3a, neutralising antibodies were detectable from week two onwards in all of the groups except for birds vaccinated with the SB-1-UL21/22VP2 vaccine. However, neutralising antibodies against IBDV were detected in the sera of all the birds in the four groups at 4 weeks post-vaccination (Fig. 3a), with no significant differences between the groups in the mean antibody levels.

**Fig. 4:**
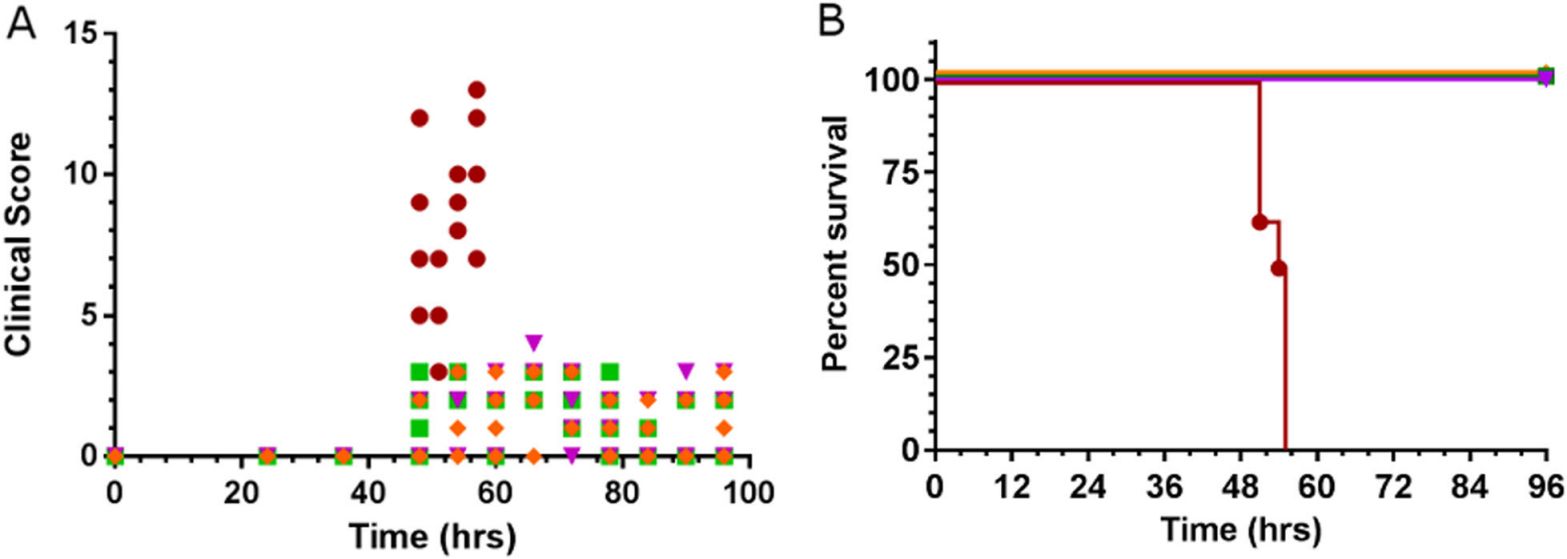
**a** Clinical score in birds vaccinated with VAXXITEK_HVT+IBD_, SB-1-UL3/4VP2, SB-1-UL21/22VP2 and SB-1 following infection with10^4.3^ TCID_50_ of IBDV strain UK661. Clinical scores are shown for individual birds. A rapid increase in the clinical score after 45 h post challenge was observed in SB-1 group and increased to its highest level at 55 h post challenge when affected birds were euthanized for humane reasons. **b** Percentage survival for the vaccinated birds challenged with IBDV UK661. Birds inoculated with pSB-1 were euthanized for humane reasons or died 55 h post IBDV infection. VAXXITEK_HVT+IBD_ (), SB-1(), SB-1-UL3/4VP2 (), SB-1-UL21/22VP2 ()

Further evaluation of the recombinant SB-1 vaccine to induce protection against IBD was carried out in Experiment 2 using an IBDV challenge model by infecting with very virulent UK661 strain at 4 weeks post-vaccination. Pre-challenge serum samples collected 3-weeks after vaccination and analysed for the presence of neutralising antibodies. The results are shown in Fig. 3b. It was shown that in groups VAXXITEK_HVT+IBD_ and SB-1-UL3/4VP2 one bird in each group and three birds in SB-1-UL21/22VP2 group had neutralising antibodies (Fig. 3b). Infection by intranasal administration of 10^4.3^ TCID_50_ of IBDV strain UK661 induced clinical signs only in birds inoculated with the control pSB-1 virus lacking VP2. The mean clinical scores of the disease in the different groups of birds during the post-challenge period are shown in Fig. 4. Clinical signs seen in the pSB-1 control group appeared from about 36 h post challenge and increased sharply until 56 h post challenge (Fig. 4a). At this time, the last remaining bird was euthanized before reaching the humane end point. All of the vaccinated birds survived (Fig. 4b), demonstrating clinical protection against the disease (*p* < 0.0001).

#### Gene expression analysis

**Fig. 5:**
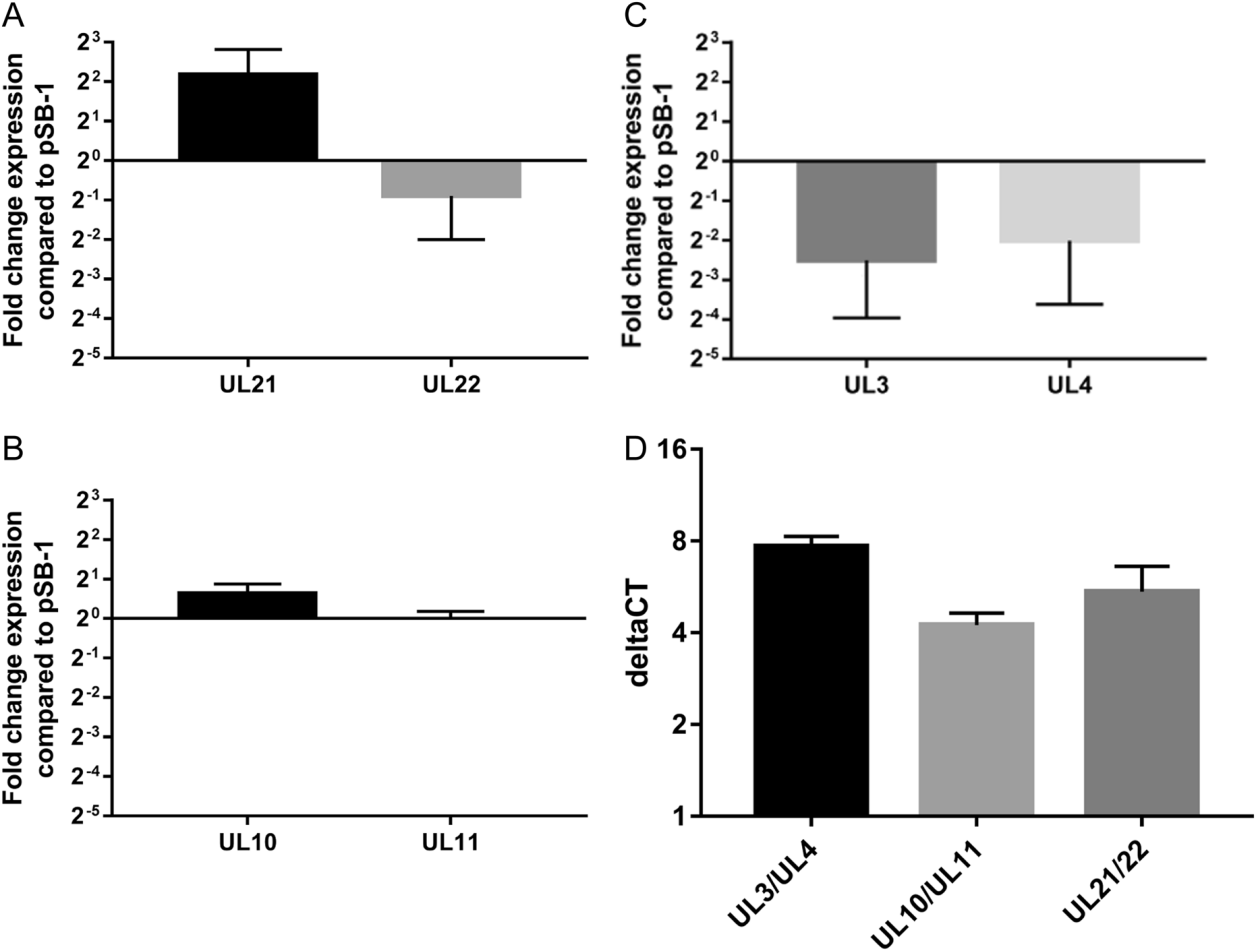
Gene expression analysis for **a** UL21 and UL22 in cells infected with SB-1-UL21/22VP2, **b** UL10 and UL11 in cells infected with SB-1-UL10/11VP2, and **c** UL3 and UL4 in cells infected with SB-1-UL3/4VP2 (**c**). Fold change expression was presented as 2^−ΔΔCt^ on the *Y*-axis of each graph. Expression of VP2 from the 3 insertion loci are shown (**d**) data are presented as ΔCt on the *Y*-axis. The groups are shown on the X axis. A significant difference in the expression of VP2 was observed in cells which were infected with SB-1-UL3/4VP2 compared to the cells infected with SB-1-UL21/22VP2 or SB-1-UL10/11VP2 (*p* = 0.0298 or *p* = 0.0035, respectively)

A SYBR green real-time PCR based assay was designed to study the level of expression of viral genes adjacent to the VP2 expression cassette. According to our data, in SB-1-UL21/22VP2, the expression level of UL21 was increased by about 4 folds; whereas the expression level of UL22 dropped by 2 folds (Fig. 5a). In cells infected with the SB-1-UL10/11VP2 virus, the level for both UL10 and UL11 transcripts were modestly increased (Fig. 5b). Finally, in cells infected with SB-1-UL3/4VP2 infected cells (Fig. 5c), level of UL3 and UL4 transcripts were dropped by about 4 folds.

In addition to the expression level for the genes adjacent to the VP2 cassette, we have calculated the level of expression for VP2 in the cells infected with the three vaccine vectors (Fig. 5d). Figure 5d shows, ΔCt value for expression level of VP2 normalised to the level of GAPDH. According to the results, more VP2 was expressed in CEF cells that were infected with SB-1-UL3/4VP2 compared to SB-1-UL21/22VP2 (*p* = 0.0298) or SB-1-UL10/11VP2 (*p* = 0.0035).

## Discussion

Avian herpesvirus vaccines used against poultry diseases such as MD offer the additional advantage of being developed as potential viral vectors for inducing simultaneous protection against other avian diseases. The best example of this is the commercial use of recombinant HVT^22,23^ as a successful vaccine inducing simultaneous protection against MD and IBD. While HVT is a highly efficient recombinant vector that could be used to induce strong immune response to a number of avian pathogens, widespread usage of HVT-vectored vaccines is hampered by the apparent interference between the recombinant viruses, for reasons that remain poorly understood. Recombinant vaccines based on other avian herpesvirus vaccine strains such as MDV-1 have been experimentally shown to be effective against diseases such as IBD, but are not yet commercially available.^24,25^ Considering the need for protecting poultry against multiple pathogens, there is the need for additional vector platforms that can deliver protective antigens without interference with the herpesvirus vaccines used in poultry.

In this study, we report the use of MDV-2 (GaHV3) strain SB-1 as a viral vector, generating three independent constructs that express IBDV VP2 in one of three different locations in the viral genome: loci UL3/4, UL10/11 and UL21/22. The expression of the VP2 cassette from the three loci appeared to slow the growth of SB-1 in vitro. Among the three locations, the VP2 expressing transcripts were appeared to have a higher level of expression from the UL3-UL4 location compared to UL21-UL22 or UL10-UL11 loci. On the other hand, level of expression for the adjacent genes was shown to be least affected when the transgene was expressed from the UL10-UL11 location.

Immunogenicity of the recombinant SB-1 vaccines was assessed by measuring the neutralising antibody levels in vaccinated chickens. Neutralising antibodies started to appear from week 2 and rose to a maximum titre of 640 in week four post-vaccination. All the birds in all four groups showed neutralising antibodies by 4 weeks. Although not statistically significant, the mean values of neutralising antibodies in the groups inoculated with the experimental vaccines were higher than those of the group that received the commercial vaccine. In the second experiment, we examined the effect of the SB-1 vaccines administered at a reduced dose of 1000 plaque forming units (pfu), compared to the 3000–5000 pfu dose of VAXXITEK_HVT+IBD_ given in both experiments. In comparison with experiment 1, only a very limited number of birds showed neutralising antibodies at week 3 post vaccination, presumably reflecting the smaller dose given. In spite of this, all of the groups showed 100% protection against IBDV after an experimental challenge with the very virulent UK661 strain of IBD virus, compared to the group vaccinated with the parental pSB-1 vaccine that not express the VP2 antigen. This suggests that the mechanism of protection may not be solely mediated by high titres of serum neutralising antibodies, and that cellular or mucosal responses may also contribute. However an evaluation of the mechanism of protection is beyond the scope of this study. Moreover, demonstration of protective responses elicited by the recombinant SB-1 vaccine against very virulent IBDV infection when used at levels comparable to the widely used VAXXITEK_HVT+IBD_ vaccine confirms the value of SB-1 as a recombinant vaccine vector platform for avian diseases.

MDV and IBDV are highly infectious viruses producing diseases with high mortality rates that have made them a constant threat to the productivity of the worldwide poultry industry for decades. The presence of maternal antibodies, emergence of new antigenic and pathogenic variants, cost of production, and in some cases lack of compliance with DIVA strategy (reviewed in^26^) are challenges that limit the efficient control of IBDV, while MDV vaccines have been demonstrated to drive virulence of the pathogen over the last 50 years.^26^ Bivalent MDV/IBDV vaccines allow for vaccination against both diseases simultaneously, lowering the costs of production and inoculation. They are also safer and can be given as in ovo vaccination, unlike attenuated IBDV vaccines that cause subclinical IBD in chicks and are fatal to embryos. While the VAXXITEK_HVT+IBD_ vaccine based on the HVT vector is widely used as a successful vaccine against IBD in many countries, there is constraint on the use of this recombinant vaccine in combination with other HVT-based vaccines, because of interference with the induction of immune responses against the components of other vaccines. Although the mechanisms of such interference between HVT-based vaccines still remain unclear, there is a definite need for other vector platforms that can complement the HVT-based vectors in multivalent vaccines.

In this paper we show that a GaHV3 (MDV-2) strain SB-1, already in use as a licensed vaccine against MD since the mid-1980s, can be used as a viral vector expressing the IBDV VP2 capsid antigen to induce complete protection against a challenge with a 100% lethal dose of IBDV. We have identified three locations in the SB-1 genome that tolerated the VP2 expression cassette, and we predict that these loci will also support the delivery of genes from other pathogens as well. With a long history of successful use with HVT as bivalent vaccine against MD, SB-1 has not shown any interference in inducing immune responses against MD. On the contrary, SB-1/HVT bivalent vaccines do provide superior protection against MD than either vaccine used alone, through synergistic effects.^2,27^ Our results demonstrating the potential of the recombinant SB-1 to protect against IBD offers immense opportunities for its use as a bivalent vaccine together with recombinant HVT to induce simultaneous protection against multiple avian diseases, exploiting their enhanced synergistic immune functions.

## Materials and methods

### Cloning of IBDV VP2 expressing cassette into SB-1 virus genome

We have used the pSB-1 BAC clone^14^ to generate three different recombinant viruses pSB-1-UL3/4VP2, pSB-1-UL10/11VP2 and pSB-1-UL21/22VP2 that express the IBDV VP2 expression cassette in the UL3/4, UL10/11 and UL21/22 intragenic loci of the SB-1 virus genome. The location of insertion sites are given in Table 1. Methods for the *galK* selection-based recombineering approach have been previously described.^16,28^ Briefly, a *galK* expression cassette was inserted into the three locations and positive colonies were selected based on their ability to utilise galactose as the sole carbon source in a minimal media. The *galK* cassette was then replaced by the VP2 expression cassette amplified from the recombinant HVT expressing IBDV VP2.^5^ Positive colonies were selected based on their ability to grow in the presence of 2-deoxy-galactose^28^ and the integration of VP2 cassette was confirmed by specific PCR and sequencing.

**Table 1:** Insertion locations for the VP2 expression cassette in SB-1 genome

Location	Insertion site
UL3/4	GATCGAC_19265_- T_19266_CGCTTTC
UL10/11	CTAAATCT_32130_-A_32131_CAAGTG
UL21/22	GTATGTG_49807_-C_9808_CTCTACAG

Numbers correspond to the genomic location of *Gallid* herpes virus—3 (SB-1) genome (GenBank accession number AB049735.1)

### Cell culture and virus propagation

Chicken embryonic fibroblasts (CEF) were prepared from 9–10 day old embryos of specific-pathogen-free (SPF) Rhode Island Red (RIR) birds in E199 media (Sigma) with 5% foetal bovine serum. DF-1 cells were propagated in Dulbecco’s modified Eagles medium (DMEM, Sigma) with 10% serum. DT40 cells were propagated in RPMI-1640 medium with 10% serum. All of the cell culture media were supplemented with 100 U/ml penicillin, 100 μg/ml streptomycin and 0.25 μg/ml fungizone.

For the preparation of recombinant virus stocks, CEF were transfected with the BAC DNA from the recombinant constructs using Lipofectamine® transfection reagent (ThermoFisher) and reconstituted viruses were passaged to generate working virus stocks. Titration of SB-1 vaccine viruses was performed in CEF and the titres calculated by counting the plaque numbers four days post-infection. Recombinant virus plaques were confirmed using immunostaining with IBDV VP2-specific mouse monoclonal antibody clone HH7 (IgG1)^29^ followed by staining with goat anti-mouse HRP conjugated antibody (DAKO) and TrueBlue™ (KPL) peroxidase substrate. Furthermore, CEF infected cells were stained with monoclonal antibodies IA7 (anti-IBDV VP2) (IgG2a) (unpublished) and SB-1-specific monoclonal antibody Y5.9.^18^ IA7 and SB-1 stained infected cells were visualised with anti IgG2a Alexa Fluor 568 and anti IgG1 Alexa Fluor 488 antibodies, respectively. Stained cells examined with a Leica SP5 confocal microscope.

Virulent IBDV UK661 strain was used as bursal tissue lysates from infected birds harvested at 3 days post infection,^30^ and the D78 strain was propagated in DF-1 cells and stored at −80 °C until use. Titrations of UK661 and D78 virus strains were performed in DT40 and DF-1 cells respectively by calculating the median tissue culture infectious dose (TCID_50_) using the Spearman-Karber method.^31^ IBDV strain D78 was used to perform virus neutralisation test using DF-1 cells.

### Virus growth curve studies

Confluent CEF in 10 cm^2^ dishes were infected in duplicate with 100 μl of 10^3^ pfu of SB-1 viruses. Following the infection, infected CEF cells were harvested at time points 0, 12, 24, 48, 96 and 120 h post infection. The harvested cells were titrated immediately by plaque assay. The experiment was repeated independently three times.^15,32,33^

### Validating the immunogenicity of vaccine viruses

One-day-old SPF RIR chicks reared at the Experimental Animal House at Pirbright Institute were used for the validation experiments. All procedures were performed in accordance with the UK Animal (Scientific Procedures) Act 1986 under Home Office Personal and Project licences, after the approval of Animal Welfare Ethical Review Board (AWERB) at The Pirbright Institute.

#### Experiment 1

Forty 1-day old chicks were divided into 4 groups of 10 birds. Each of the three groups received subcutaneous injections of pSB-1 UL10/11 VP2, pSB-1 UL21/22 VP2 or pSB-1 UL3/4 VP2 vaccine viruses, each comprising 3 × 10^3^ pfu in 100 µl of inoculum. Each of the 10 birds in the control group were vaccinated with 5 × 10^3^ pfu of the Vaxxitek_HVT+IBD_® vaccine (Merial) as recommended by the manufacturer. Blood samples were collected weekly from the second to the fifth week post vaccination for serological studies.

#### Experiment 2

Two groups (8 birds per group) of 1-day old birds were inoculated subcutaneously with 1000 pfu of pSB-1 UL3/4 VP2 or pSB-1 UL21/22 VP2 virus stocks. Two control groups were inoculated with either pSB-1-derived parental virus (1000 pfu) or the commercial Vaxxitek_HVT+IBD_® vaccine respectively. After collecting the blood samples at four week post-vaccination, birds were challenged intra-nasally with 10^4.3^ TCID_50_ of the virulent UK661 strain of IBDV (in a total volume of 100 µl divided between the two nostrils). In addition to the recording of the body weight, clinical score of birds were monitored regularly using a scoring system based on appearance, behaviour, provoked behaviour and handling (supporting materials 1). Clinical signs scored at 6-hourly intervals. Birds showing advanced clinical signs (exceeding a score of 9) or if they scored 5 for two consecutive intervals were euthanized by cervical dislocation.

### Serology

Serum samples collected by centrifugation were heat treated at 56 °C for 30 min to inactivate complement factors, prior to the neutralisation test. Briefly, serial dilutions of sera samples were incubated with 100 TCID_50_ of D78 strain of IBDV for one hour at 37 °C, and the serum-virus mixtures were incubated with DF-1 monolayer in 96 well plates for one hour, before replacing with 2% DMEM media. The cells were checked after four days for evidence of cytopathic effects to determine serum neutralisation titres. The highest dilution of the serum that prevented cytopathic effect was considered as the neutralisation titre.

### Real-time PCR assay for analysis of gene expression

Confluent CEF cells were infected with 500 pfu of SB-1 viruses in 3.45 cm diameter tissue culture plates. Infected cells were harvested 72 h post infection. The cells were washed with PBS and scraped from the plate. RNA purification was performed using RNeasy Qiagen kit. Extracted RNA was treated with DNAseI (New England Biolabs) for 30 min followed by heat inactivation at 75° to degrade any carry over DNA. Reverse transcription was performed using random primers and Revertaid reverse transcriptase (Thermo Fisher Scientific) as described by the manufacturer. SYBR green based real-time PCR assay was performed using PowerUp SYBR green master mix (Thermo Fisher Scientific). The real-time PCR reactions were assembled in 10 μl volumes and in an Applied Biosystems 7500 fast real-time-PCR system. PCR cycles were programmed as recommended by the manufacturer with annealing at 60 °C (30 s) and extension at 72 °C (30 s). The signal was collected at the extension cycle. Melt curve analysis was performed after each PCR run to eliminate the possibility of non-specific amplifications. To compare the data, ΔΔCt value was calculated for each gene of interest. To do so, the Ct value of GAPDH in each real-time PCR experiment was used as the house keeping gene. The Ct values for each of the genes studied in CEF cells infected with SB-1 parental virus was used as the calibrator. The assay was performed in three biological replicates with all of the samples were kept at −20 and analysed at the same time for RNA purification and Real-time PCR assay. SYBR green PCR primers were designed using Primer3 primer design server. They were validated by conventional PCR prior to perform the assay. PCR primers: GAPDH_SYBR_F1: actgtcaaggctgagaacgg, GAPDH_SYBR_R1: ctgcatccgcccatttgatg, UL3_F: tctcgacgaattgggaagac, UL3_R: gagcttgaattaccgcttgc, UL4_F: tcttatcggatcgcagctct, UL4_R:tggatgggaacgtcactgta, UL10_F:ggcatgattgttcgcctaat, UL10_R:tctcgtcgtctgatgtttcg, UL11_F:ccgaccgtccttaaatctga, UL11_R:aacgaaacaccgttctgacc, UL21_F:gaggggcaacttaaacacca, UL21_R:caattcccgcaactccttta, UL22_F:ccgcaatacggacattcttt, UL22_R:aatgttcgggcactgatagg, VP2_F: cttccaaggaagcctgagtg and VP2_R: tgtcactgctgtcgcatgta.

### Statistical analysis

Two way ANOVA with multiple comparisons test was employed to compare virus titres at each time point of the growth curve. Differences in levels of neutralising antibody during the course of study and between the groups were analysed using two-way ANOVA test. The level of antibodies within each group was analysed using one-way ANOVA test. The survival rate between groups of the birds after the challenge was compared using the Mantel-Cox test.

## Electronic supplementary material

Supporting document 1
