# Supplementary material for: Gallid herpesvirus 3 SB-1 strain as a recombinant viral vector for poultry vaccination
Source: NPJ Vaccines. 2018 May 28;3:21. doi: 10.1038/s41541-018-0056-6 (PMC5972151; doi:10.1038/s41541-018-0056-6)
Supplement: Supplementary file 1 — Supporting document 1 [file 41541_2018_56_MOESM1_ESM.pdf]

IBDV specific clinical response:
The main clinical signs of IBDV infection are watery diarrhoea, ruffled feathers, reluctance to move, anorexia, trembling and prostration. A flock will show very high morbidity with severe depression in most birds lasting for 5–7 days. Mortality rises sharply for 2 days then declines rapidly over the next 2–3 days. The clinical scoring system of the Project licence will be used (see below).

Any adverse effects seen by EAH staff will be conveyed asap to laboratory staff by either phone and / or e-mail.
PLEASE NOTE: THE SEVERITY OF THIS REQUEST IS MODERATE. IF A SCORE OF +5 IS REACHED FOR 2 CONSEQUTIVE DAYS THEN BIRDS WILL BE KILLED.

**A) Appearance**	**Score**
Normal	0
Pale comb / wattles	1
Dishevelled / moderately fluffed out feathers	1
Very fluffed out feathers	2
Very fluffed out feathers and half shut eyes	3
Drooping wings/ hunched / hangs head	4

**B) Behaviour**	**Score**
Normal posture	0
Huddles with group	2
Tends to stand down alone	4
Stands / sits alone all the time	E (kill by schedule 1 method)

**C) Provoked Behaviour**	**Score**
Normal / escapes capture	0
Slight impairment in response to capture	2
Weak response to capture	4
Fails to move when prodded	E (kill by schedule 1 method)

**D) Handling**	**Score**
Feels normal	0
Crop empty (Not feeding or drinking)	2
Soiled vent feathers	3
Very thin / keel feels very pronounced	4

If total score is 0-5 for ≤ 2 days severity is deemed mild and birds are to be inspected normally.
If total score is +5 for > 2 days severity is deemed moderate and birds are to be killed by a schedule 1 method.
